# Versatility of Impella^®^ Ventricular Assist Devices in High-risk Cardiac Patients During Complex Procedures: A Case Series

**DOI:** 10.19102/icrm.2024.15113

**Published:** 2024-11-15

**Authors:** Nagaraj Swaminathan, Madison Hazelwood, Nadine Odo, Mallikarjuna R. Devarapalli

**Affiliations:** 1 Department of Anesthesiology and Perioperative Medicine, Medical College of Georgia, Augusta University, Augusta, GA, USA

**Keywords:** Atrial fibrillation, cardiac catheterization, electrophysiology, heart-assist devices, hemodynamics

## Abstract

High-risk cardiac patients undergoing complex electrophysiology procedures face potential hemodynamic instability, necessitating effective mechanical circulatory support. The catheter-based Impella^®^ ventricular assist device (Abiomed, Danvers, MA, USA) is crucial to stabilizing hemodynamics by improving the flow of blood from the left ventricle to the aorta. Its automated controller ensures cerebral hemodynamic stability, allowing for bedside adjustments. Herein, we present a case series illustrating the versatility of the Impella^®^ device in managing patients during complex electrophysiology procedures and highlighting its role in mitigating hemodynamic compromise.

## Background

Complex electrophysiology (EP) procedures in high-risk cardiac patients pose challenges due to potential hemodynamic instability.^1,2^ Mechanical circulatory support (MCS) during such procedures plays a pivotal role in maintaining native cardiac function.^3–6^ Impella^®^ (Abiomed, Danvers, MA, USA), a catheter-based miniature ventricular assist device, stands out among MCS devices for its ability to provide mechanical hemodynamic stabilization by augmenting cardiac output.^7^ The Impella^®^ device generates mechanical flow, allowing for hemodynamic stabilization of patients undergoing complex high-risk procedures by aspirating oxygenated blood from the left ventricle (LV) and expelling it into the aorta. Its real-time monitoring capabilities via the Automated Impella^®^ Controller offer flexibility and precision, making it an optimal choice for select high-risk cases.^8–11^

During the procedure, Impella^®^ 2.5 or Impella^®^ CP with SmartAssist is placed across the aortic valve. An external console monitors the pressure difference between the LV and aorta and permits adjustment of the motor speed to up to 2.5–5.0 L/min to stabilize cerebral hemodynamics. Thus, Impella^®^ can be an effective alternative to MCS systems like the intra-aortic balloon pump, venoarterial extracorporeal membrane oxygenation, and TandemHeart^®^ (Cardiac Assist, Inc., Pittsburgh, PA, USA).^11,12^

We describe the utility of the Impella^®^ device during three distinct complex EP procedures in which it facilitated (1) accurate mapping of arrhythmias, (2) management of unforeseen complications during a pulmonary vein isolation (PVI) procedure, and (3) prevention of cardiogenic shock following implantation of a Watchman FLX™ device (Boston Scientific, Marlborough, MA, USA).

The patients provided written Health Insurance Portability and Accountability Act authorization for the publication of this case series, as per institutional policy.

## Case presentations

### Case 1

**Figure 1:**
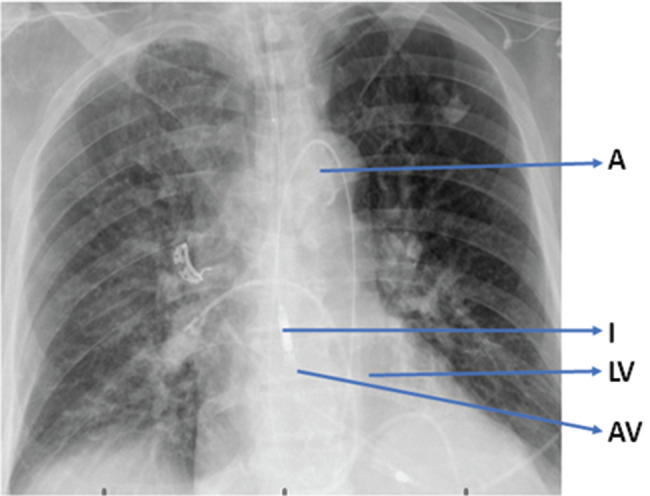
Chest X-ray of the Impella^®^ device lying across the aortic valve into the left ventricle. *Abbreviations:* A, aorta; AV, approximate position of the aortic valve; I, Impella^®^; LV, left ventricle.

A 77-year-old man with a history of non-sustained ventricular tachycardia (VT), atrial fibrillation (AF), congestive heart failure, chronic kidney disease, and low ejection fraction (EF) presented with recurrent shocks from his implantable cardioverter-defibrillator, indicative of a VT storm. Despite medical therapy, including amiodarone and mexiletine, the patient continued to experience VT episodes, necessitating hospitalization. VT storm episodes continued after unsuccessful treatment with flecainide and VT ablation surgery; therefore, the patient underwent a repeat ablation. Because the initial ablation procedure by activation mapping in the endocardial regions was unsuccessful, the electrophysiologist deduced that the focus of triggered activity was likely from the epicardium and therefore mapped the epicardial region. As epicardial access can result in hemodynamic compromise, an Impella^®^ 2.5 device was used for hemodynamic support **(Figure 1)**. Triggered activity originating from an inferior wall scar was detected. The ablation targeted the critical isthmus, originating from the apex of the anterior epicardium of the LV. Additional minor VT sources identified through entrainment were also ablated. The patient was rendered hemodynamically stable with the inotropic agent epinephrine. The MCS device was subsequently removed, and the patient was transferred to the intensive care unit for monitoring.

### Case 2

A 77-year-old man with AF, hyperlipidemia, and a coronary stent replacement was initially scheduled for a PVI ablation procedure; however, a left atrial appendage (LAA) thrombus was detected on the preoperative computed tomographic angiogram. The procedure was postponed, and the patient’s dosage of apixaban was increased for 2 weeks. A transesophageal echocardiogram (TEE) recorded before the rescheduled PVI verified resolution of the LAA thrombus. However, during the procedure, the patient exhibited acute ST-segment elevation in V1–V3, QRS widening of 140 ms, ventricular ectopy, and prolonged non-sustained VT, resulting in hypotension. Prompt stabilization was achieved with advanced cardiovascular life support, including administration of amiodarone, epinephrine, and cardiopulmonary resuscitation. Urgent cardiac catheterization revealed a proximal left anterior descending thrombus, prompting aspiration thrombectomy and balloon angioplasty. Despite pharmacological and inotropic support, the patient remained hypotensive; therefore, an Impella^®^ CP device was used to supplement hemodynamics and ensure adequate cerebral perfusion. The patient was subsequently transferred to the cardiovascular intensive care unit for further observation and management.

### Case 3

A 68-year-old woman with a history of paroxysmal AF, chronic kidney disease, and recurrent gastrointestinal bleeding opted for the implantation of a Watchman FLX™ device to discontinue anticoagulation therapy and address bleeding issues. After extubation, the patient developed an acute rapid ventricular response with acute respiratory distress. The patient was cardioverted three times without a response. Non-invasive ventilation provided temporary stabilization as she was transferred to the postanesthesia care unit. The patient was re-intubated due to hemodynamic compromise. Evidence of a posterior ST-segment–elevation myocardial infarction on electrocardiography prompted immediate intervention in the catheterization laboratory. Balloon angioplasty was used to restore blood flow, as angiography revealed acute occlusion of the mid-left circumflex artery. Despite successful revascularization, the patient developed VT, necessitating rapid hemodynamic stabilization.

As the patient was not responding to inotropic drugs and, given her complex cardiac condition and recent surgical intervention, an Impella^®^ CP device was used for hemodynamic stability and cerebral perfusion. The patient was transported to the cardiac care unit with Impella^®^ support. The MCS was later removed, enabling the patient to return home.

## Discussion

### Perioperative management strategies

The care of patients undergoing Impella^®^ support involves several important perioperative considerations. Echocardiography is crucial to assessing right ventricular function, monitoring for device-related complications, and confirming device positioning.^13^ In our practice, we commonly use TEE combined with fluoroscopy during implantation procedures. TEE ensures correct placement of the guidewire within the aortic lumen across the aortic valve, reaching the LV with its tip oriented toward the apex, which is often visualized through the mid-esophageal long-axis view.^14^ Furthermore, it is essential to use transthoracic echocardiography to verify central placement within the ventricle and assess for potential injury to the mitral valve, aortic valve, or aorta, which can mitigate the risk of arrhythmias and suction events.^14^ Once the MCS is appropriately positioned, the gradual initiation of pump flow allows the right ventricle to adapt to the increased preload, and color Doppler can confirm flow from the LV into the aorta through the inlet and outlet.^14^

Alongside intracardiac echocardiography, pulmonary artery catheterization and arterial blood pressure monitoring play critical roles in optimizing the management of patients supported by the Impella^®^ device.^13^ In retrospective analyses of registry data, pulmonary artery catheter monitoring for hemodynamic assessment before and during transvalvular heart pump support was associated with improved survival outcomes, making it integral to perioperative evaluation.^14^ To prevent an inadequate preload and ventricular dysfunction, it is essential to monitor for changes in ventricular size and dilatation, pulmonary capillary wedge pressure (PCWP), contractility, interventricular septal shifts, and mitral regurgitation.^13^ During device placement, heparin infusion is standard to achieve a target clotting time of 250 s, reducing the risk of intraoperative thrombosis.^14^

Our patients presented with multiple cardiovascular comorbidities and significantly reduced EFs, posing increased anesthesia risks. Additionally, the presence of ventricular arrhythmias, possibly related to sarcoidosis or coronary artery disease, led to unique challenges. Patients with low EFs often cannot tolerate complex, high-risk procedures, necessitating Impella^®^ placement to provide circulatory support and maintain cerebral perfusion. Stimulation of the heart with a low EF can lead to poorly tolerated responses, including asystole; thus, LV assist device implantation can help to maintain cerebral perfusion even during compromised systemic perfusion. In the first case, the EP team and invasive cardiologist opted for Impella^®^ device insertion due to the patient’s vulnerability to hemodynamic instability because of ischemic cardiomyopathy and malignant arrhythmias. This patient, who was refractory to medications and implantable cardioverter-defibrillator therapy, underwent ventricular catheter ablation.

Common postoperative sequelae can be predicted and managed to optimize patient outcomes. Fluid overload resulting from intraoperative blood transfusions is frequent, often necessitating diuretic therapy. This is especially true when post-procedure red blood cell transfusions are required due to blood loss. Monitoring PCWP and central venous pressure (CVP) helps assess the fluid status; elevated PCWP and CVP with a normal cardiac index indicates potential fluid excess, while low PCWP and CVP may suggest low intravascular volume.^14^ Cardiogenic shock, a life-threatening complication of EP ablation, can be managed with various pharmacologic agents to enhance inotropy, vascular tone, and fluid administration.

Multiple attempts to increase the P-level of Impella^®^ from P4 to P5 or P6 to promote greater perfusion were not tolerated, instead resulting in a suction event, suggesting that P4 may be optimal in similar patients, and caution should be exercised when manipulating the P-level postoperatively. In general, a pulsatility that brings the cardiac index, CVP, and PCWP within normal ranges is used, and significantly increased PCWP may indicate greater ventricular support.^14^

Transitioning patients off Impella^®^ support typically begins when the mean arterial pressure reaches 65 mmHg, the heart rate is <100 bpm, and there are moderate levels of device pulsatility alongside pharmacologic stability, indicating improved perfusion (lactate concentration < 2 mmol/L).^14^ In our case, we successfully removed the Impella^®^ device by gradually reducing the pulsatility flow with the aid of epinephrine and vasopressors to support perfusion and facilitate device removal. Monitoring Impella^®^ removal without echocardiographic and pulmonary artery catheter assistance confirmed stable cardiac function, as reflected by the relatively unchanged cardiac index and output post-removal.

Frequent imaging, including echocardiography and X-rays, remains crucial to assess post-procedure systolic function, detect pericardial effusion, and identify atelectasis. Dyspnea after Impella^®^ procedures can have multiple causes, such as exacerbation of cardiogenic shock, pericardial effusion, or atelectasis, and may require intubation if refractory to oxygen therapy. Attention to renal function is essential due to potential cardiogenic and hypovolemic shock-induced diminished perfusion and metabolic acidosis. Monitoring for sepsis and gastrointestinal complications is also imperative. Aggressive management of short-term sequelae ensures favorable outcomes, allowing for timely discharge without long-term complications.

### Literature review and conclusion

Case 1 demonstrates the unique role of the Impella^®^ device in a patient whose prior surgical intervention could complicate the identification of the origin of arrhythmias. It is imperative that appropriate LV unloading is achieved to accurately map the critical isthmus of the arrhythmias. Impella^®^ achieves this by facilitating end-organ perfusion during periods of sinus rhythm, thus reducing the myocardial oxygen demand. However, the use of Impella^®^ in high-risk populations has been associated with an increased arrhythmic burden. Therefore, it is important to counsel patients, especially those with multiple comorbidities, on the increased mortality associated with its use in arrhythmia ablation and repair.^14,15^ Furthermore, this case suggests that, with these devices, adjustable hemodynamic support can be achieved when pacemaker placement is not possible.^14,16^

Case 2 suggests the value of the Impella^®^ device in managing acute cardiogenic shock (ACS). While there are no specific guidelines for Impella^®^, this case provides valuable insight that may influence operative and postoperative outcomes in the setting of ACS.^17^ The Prospective, Randomized Clinical Trial of Hemodynamic Support with Impella^®^ 2.5 Versus Intra-Aortic Balloon Pump in Patients Undergoing High-risk Percutaneous Coronary Intervention (PROTECT II) trials indicate that Impella^®^ can improve revascularization and LV function. This was further supported by hemodynamic studies by Schäfer et al.^17^ Evidence from the literature and our case points toward the efficacy of Impella^®^ devices in ACS.

Lastly, case 3 highlights the need for appropriate preventative measures to avoid cardiogenic shock during a Watchman FLX™ procedure secondary to device-related thrombosis. The Impella^®^ not only assists with continuous hemodynamic stabilization but also provides support in cases of cardiogenic shock, a common complication of such procedures. The Watchman FLX™ vs. Watchman 2.5 in a Dual-Center Left Atrial Appendage Closure Cohort (WATCH-DUAL) study suggested the incidence of post-procedural cardiovascular death to be as high as 3.1%.^18^ There are additional 1.6% and 1.4% risks of cerebrovascular events and acute kidney injury, respectively, associated with this procedure.^12^ To date, there have been no studies on the use of Impella^®^ in these procedures. However, decreasing LV strain and improving end-organ perfusion could potentially improve outcomes in these patients.

While Impella^®^ is an efficient MCS device, it does come with certain drawbacks. Compared to other MCS devices, such as the intra-aortic balloon pump, the Impella^®^ device is inserted using a larger catheter, thereby increasing the risk of arterial ischemia and infection due to a larger vessel and soft tissue insult.^18^ Additionally, as studies of Impella^®^ are limited, guidelines that consider a patient’s comorbidities as well as postoperative device-related mortality are needed.^18^ We hope that by showcasing our management of these cases, we may be able to move toward the incorporation of the Impella^®^ device in a standardized fashion across multiple EP cardiac procedures.
